# Supplementary material for: Modeling glioblastoma heterogeneity as a dynamic network of cell states
Source: Mol Syst Biol. 2021 Sep 16;17(9):e10105. doi: 10.15252/msb.202010105 (PMC8444284; doi:10.15252/msb.202010105)
Supplement: Supplementary file 6 — Source Data for Figure 5 [file MSB-17-e10105-s004.zip › Figure5A_sourcedata/GSEA_3071/hallmarks_stateV.GseaPreranked.1623742546329/HALLMARK_DNA_REPAIR.html]

Details for gene set HALLMARK\_DNA\_REPAIR[GSEA]

|  || Dataset | state43071 |
| Phenotype | NoPhenotypeAvailable |
| Upregulated in class | na\_pos |
| GeneSet | HALLMARK\_DNA\_REPAIR |
| Enrichment Score (ES) | 0.39845482 |
| Normalized Enrichment Score (NES) | 2.0061746 |
| Nominal p-value | 0.0 |
| FDR q-value | 6.305024E-4 |
| FWER p-Value | 0.01 |
Table: GSEA Results Summary

  

Fig 1: Enrichment plot: HALLMARK\_DNA\_REPAIR      
 Profile of the Running ES Score & Positions of GeneSet Members on the Rank Ordered List

  

| PROBE | GENE SYMBOL | GENE\_TITLE | RANK IN GENE LIST | RANK METRIC SCORE | RUNNING ES | CORE ENRICHMENT || 1 | PCNA |  |  | 10 | 1.908 | 0.0415 | Yes |
| 2 | TYMS |  |  | 30 | 1.683 | 0.0762 | Yes |
| 3 | POLD3 |  |  | 111 | 1.340 | 0.0914 | Yes |
| 4 | FEN1 |  |  | 139 | 1.296 | 0.1158 | Yes |
| 5 | LIG1 |  |  | 145 | 1.284 | 0.1441 | Yes |
| 6 | RFC4 |  |  | 171 | 1.227 | 0.1672 | Yes |
| 7 | ZWINT |  |  | 204 | 1.166 | 0.1877 | Yes |
| 8 | ARL6IP1 |  |  | 212 | 1.158 | 0.2127 | Yes |
| 9 | RFC3 |  |  | 239 | 1.130 | 0.2334 | Yes |
| 10 | SAC3D1 |  |  | 252 | 1.118 | 0.2566 | Yes |
| 11 | PRIM1 |  |  | 293 | 1.074 | 0.2734 | Yes |
| 12 | DUT |  |  | 314 | 1.050 | 0.2935 | Yes |
| 13 | RFC2 |  |  | 337 | 1.025 | 0.3126 | Yes |
| 14 | RPA3 |  |  | 395 | 0.953 | 0.3234 | Yes |
| 15 | POLA1 |  |  | 463 | 0.900 | 0.3311 | Yes |
| 16 | RAD51 |  |  | 522 | 0.851 | 0.3394 | Yes |
| 17 | RFC5 |  |  | 527 | 0.849 | 0.3580 | Yes |
| 18 | COX17 |  |  | 666 | 0.766 | 0.3491 | Yes |
| 19 | GPX4 |  |  | 701 | 0.746 | 0.3595 | Yes |
| 20 | POLH |  |  | 807 | 0.697 | 0.3553 | Yes |
| 21 | NME4 |  |  | 819 | 0.692 | 0.3690 | Yes |
| 22 | ALYREF |  |  | 847 | 0.680 | 0.3793 | Yes |
| 23 | DDB2 |  |  | 872 | 0.669 | 0.3899 | Yes |
| 24 | POLD1 |  |  | 998 | 0.620 | 0.3802 | Yes |
| 25 | EIF1B |  |  | 1000 | 0.620 | 0.3941 | Yes |
| 26 | POLA2 |  |  | 1161 | 0.574 | 0.3766 | Yes |
| 27 | POLR2D |  |  | 1220 | 0.556 | 0.3781 | Yes |
| 28 | AK3 |  |  | 1243 | 0.548 | 0.3864 | Yes |
| 29 | DGCR8 |  |  | 1246 | 0.547 | 0.3985 | Yes |
| 30 | REV3L |  |  | 1415 | 0.500 | 0.3777 | No |
| 31 | UMPS |  |  | 1448 | 0.493 | 0.3828 | No |
| 32 | NELFCD |  |  | 1530 | 0.474 | 0.3781 | No |
| 33 | SSRP1 |  |  | 1634 | 0.451 | 0.3687 | No |
| 34 | PDE4B |  |  | 1660 | 0.444 | 0.3740 | No |
| 35 | USP11 |  |  | 1716 | 0.434 | 0.3734 | No |
| 36 | CANT1 |  |  | 1873 | 0.407 | 0.3528 | No |
| 37 | ADCY6 |  |  | 2018 | 0.385 | 0.3341 | No |
| 38 | VPS37B |  |  | 2069 | 0.377 | 0.3331 | No |
| 39 | XPC |  |  | 2211 | 0.361 | 0.3143 | No |
| 40 | POLR2I |  |  | 2316 | 0.347 | 0.3024 | No |
| 41 | HPRT1 |  |  | 2404 | 0.334 | 0.2933 | No |
| 42 | TAF10 |  |  | 2436 | 0.330 | 0.2949 | No |
| 43 | RPA2 |  |  | 2570 | 0.314 | 0.2766 | No |
| 44 | RRM2B |  |  | 2709 | 0.298 | 0.2570 | No |
| 45 | SF3A3 |  |  | 3242 | -0.258 | 0.1612 | No |
| 46 | TAF12 |  |  | 3425 | -0.286 | 0.1329 | No |
| 47 | TMED2 |  |  | 3509 | -0.301 | 0.1239 | No |
| 48 | NME1 |  |  | 3615 | -0.317 | 0.1111 | No |
| 49 | POLR3C |  |  | 3628 | -0.320 | 0.1160 | No |
| 50 | NUDT9 |  |  | 3673 | -0.326 | 0.1151 | No |
| 51 | POLR1C |  |  | 3674 | -0.327 | 0.1225 | No |
| 52 | POLR3GL |  |  | 3676 | -0.327 | 0.1297 | No |
| 53 | GTF2B |  |  | 3682 | -0.328 | 0.1363 | No |
| 54 | ADRM1 |  |  | 3834 | -0.355 | 0.1155 | No |
| 55 | SDCBP |  |  | 3840 | -0.357 | 0.1226 | No |
| 56 | SUPT4H1 |  |  | 3950 | -0.377 | 0.1104 | No |
| 57 | POLR1D |  |  | 3981 | -0.384 | 0.1134 | No |
| 58 | NT5C3A |  |  | 4190 | -0.435 | 0.0835 | No |
| 59 | RALA |  |  | 4332 | -0.483 | 0.0676 | No |
| 60 | TAF9 |  |  | 4590 | -0.580 | 0.0317 | No |
| 61 | POLD4 |  |  | 4705 | -0.646 | 0.0246 | No |
| 62 | GTF2H5 |  |  | 4769 | -0.691 | 0.0283 | No |
| 63 | BCAM |  |  | 4776 | -0.696 | 0.0430 | No |
| 64 | ITPA |  |  | 4830 | -0.738 | 0.0496 | No |
| 65 | DAD1 |  |  | 4860 | -0.763 | 0.0615 | No |
| 66 | ADA |  |  | 5005 | -0.945 | 0.0554 | No |
Table: GSEA details [plain text format]

  

Fig 2: HALLMARK\_DNA\_REPAIR: Random ES distribution      
 Gene set null distribution of ES for **HALLMARK\_DNA\_REPAIR**
